# Challenges and advances in clinical applications of mesenchymal stromal cells

**DOI:** 10.1186/s13045-021-01037-x

**Published:** 2021-02-12

**Authors:** Tian Zhou, Zenan Yuan, Jianyu Weng, Duanqing Pei, Xin Du, Chang He, Peilong Lai

**Affiliations:** 1 Department of Hematology, Guangdong Provincial People’s Hospital, Guangdong Academy of Medical Sciences, Guangzhou, 510080 People’s Republic of China; 2 State Key Laboratory of Ophthalmology, Zhongshan Ophthalmic Center, Sun Yat-Sen University, Guangzhou, 510060 People’s Republic of China; 3 Department of Hepatic Surgery and Liver Transplantation Center, The Third Affiliated Hospital of Sun Yat-Sen University, Guangzhou, 510630 People’s Republic of China; 4 Guangdong Provincial Key Laboratory of Stem Cell and Regenerative Medicine, South China Institute for Stem Cell Biology and Regenerative Medicine, Guangzhou Institutes of Biomedicine and Health, University of Chinese Academy of Sciences, Chinese Academy of Sciences, Guangzhou, 510530 People’s Republic of China

**Keywords:** Mesenchymal stromal cells, Clinical applications, Heterogeneity, Artificial intelligence (AI), Extracellular vesicles, COVID-19

## Abstract

Mesenchymal stromal cells (MSCs), also known as mesenchymal stem cells, have been intensely investigated for clinical applications within the last decades. However, the majority of registered clinical trials applying MSC therapy for diverse human diseases have fallen short of expectations, despite the encouraging pre-clinical outcomes in varied animal disease models. This can be attributable to inconsistent criteria for MSCs identity across studies and their inherited heterogeneity. Nowadays, with the emergence of advanced biological techniques and substantial improvements in bio-engineered materials, strategies have been developed to overcome clinical challenges in MSC application. Here in this review, we will discuss the major challenges of MSC therapies in clinical application, the factors impacting the diversity of MSCs, the potential approaches that modify MSC products with the highest therapeutic potential, and finally the usage of MSCs for COVID-19 pandemic disease.

## Background

**Fig.1:**
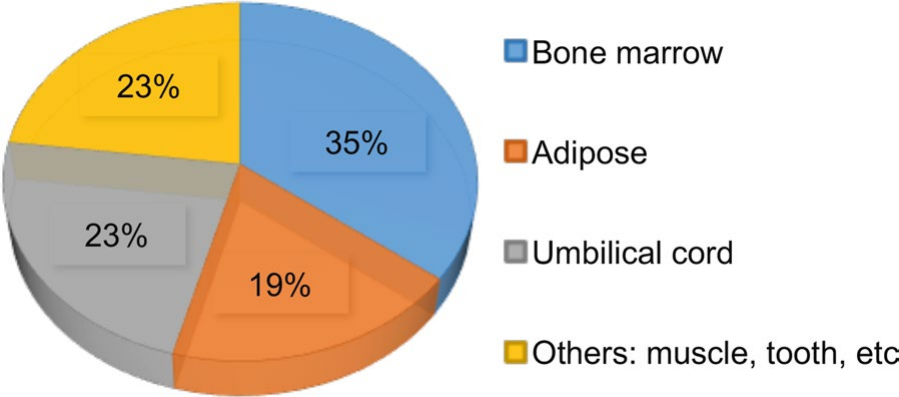
Various sources of MSCs used in the registered clinical trials. MSCs isolated from bone marrow are most widely applied in clinical trials, followed by those from umbilical cord and adipose. MSCs from muscles, tooth are also used

Mesenchymal stromal cells (MSCs) are pluripotent non-hematopoietic stem cells with self-renewal capability [1] and being intensively investigated in clinical trials. Since the discovery of MSCs from bone marrow by Friedenstein in 1970s, MSCs have been isolated from various sources including muscle, umbilical cord, liver, placenta, skin, amniotic fluid, synovial membrane, and tooth root [2, 3], and tested in amounts of preclinical and clinical studies (Fig. 1). It is now understood that MSCs have wide-ranging physiological effects including the maintenance of tissue homeostasis and regeneration [4, 5], as well as the immunomodulatory activities suitable for therapeutic application [6]. So their indications have been expanded to graft-versus-host disease (GVHD), multiple sclerosis (MS), Crohn’s disease (CD), amyotrophic lateral sclerosis (ALS), myocardial infarction (MI), and acute respiratory distress syndrome (ARDS) [7–9].

Over 300 clinical trials of MSC therapies have been completed in patients including but not limited to degenerative or autoimmune diseases (Table 1 lists some of the representative completed studies). Overall, MSCs have exhibited tolerable safety profile and demonstrated promising therapeutic benefits in some clinical settings, which led to regulatory approvals of MSCs in a few countries. In 2011, the Ministry of Food and Drug Safety (Korea FDA) approved Cartistem®, a MSC product derived from umbilical cord blood and developed by Medipost for the treatment of traumatic or degenerative osteoarthritis [10]. Thereafter, more MSC products including HeartiCellgram®, Mesoblast, TiGenix, and Stempeutics, were approved by regulatory authorities worldwide for the treatment of a variety of diseases. In the USA, Ryoncil (remestemcel-L) is promising to be the first FDA-approved GVHD treatment for children younger than 12, but is still in the stage of safety verification. The amount of clinics offering exogenous stem cell therapies has doubled from 2009 to 2014 in the USA. This boom in stem cell clinics with 351 companies putting stem cells for sale in 570 clinics in 2016 indicated the mal-practice of the MSC therapies [11]. Considering the fact that many of the applied exogenous stem cell therapies lack confirmation on safety and effectiveness from large-scale clinical trials and are even illegal, these medical mal-practices do threaten the development of MSC therapies [12].

**Table 1:** Some representative registered clinical trials of MSC therapies

NCT Number	Title	Phase	Sponsor/Collaborators
NCT02097641	Human Mesenchymal Stromal Cells For Acute Respiratory Distress Syndrome (START)	Phase 2	National Heart, Lung, and Blood Institute (NHLBI) Massachusetts General Hospital Stanford University University of Pittsburgh University of Minnesota Ohio State University University of California, San Francisco
NCT00957931	Allo-HCT MUD for Non-malignant Red Blood Cell (RBC) Disorders: Sickle Cell, Thal, and DBA: Reduced Intensity Conditioning, Co-tx MSCs	Phase 2	Stanford University University of Minnesota University of Alabama at Birmingham
NCT01771913	Immunophenotyping of Fresh Stromal Vascular Fraction From Adipose-Derived Stem Cells (ADSC) Enriched Fat Grafts	Phase 2	University of Sao Paulo
NCT01909154	Safety Study of Local Administration of Autologous Bone Marrow Stromal Cells in Chronic Paraplegia (CME-LEM1)	Phase 1	Puerta de Hierro University Hospital
NCT03102879	Encapsulated Mesenchymal Stem Cells for Dental Pulp Regeneration	Phase 1 Phase 2	Universidad de los Andes, Chile Cells for Cells, Chile
NCT02467387	A Study to Assess the Effect of Intravenous Dose of (aMBMC) to Subjects With Non-ischemic Heart Failure	N/A	CardioCell LLC Stemedica Cell Technologies, Inc
NCT02387749	Effect Of Mesenchymal Stem Cells Transfusion on the Diabetic Peripheral Neuropathy Patients	N/A	Cairo University
NCT01932164	Use of Mesenchymal Stem Cells for Alveolar Bone Tissue Engineering for Cleft Lip and Palate Patients	N/A	Hospital Sirio-Libanes
NCT02481440	Repeated Subarachnoid Administrations of hUC-MSCs in Treating SCI	Phase 1 Phase 2	Third Affiliated Hospital, Sun Yat-Sen University, China
NCT02165904	Subarachnoid Administrations of Adults Autologous Mesenchymal Stromal Cells in SCI	Phase 1	Emory University
NCT02330978	Intravitreal Mesenchymal Stem Cell Transplantation in Advanced Glaucoma	Phase 1	University of Sao Paulo
NCT01183728 NCT01586312	Treatment of Knee Osteoarthritis With Autologous/ Allogenic Mesenchymal Stem Cells	Phase 1 Phase 2	Red de Terapia Celular Fundacion Teknon, Centro Medico Teknon, Barcelona University of Valladolid
NCT02037204	IMPACT: Safety and Feasibility of a Single-stage Procedure for Focal Cartilage Lesions of the Knee	Phase 1 Phase 2	UMC Utrecht
NCT02958267	Investigation of Mesenchymal Stem Cell Therapy for the Treatment of Osteoarthritis of the Knee	Phase 2	OhioHealth
NCT00587990	Prospective Randomized Study of Mesenchymal Stem Cell Therapy in Patients Undergoing Cardiac Surgery (PROMETHEUS)	Phase 1 Phase 2	National Heart, Lung, and Blood Institute (NHLBI) Johns Hopkins University Specialized Center for Cell Based Therapy The Emmes Company, LLC University of Miami
NCT01385644	A Study to Evaluate the Potential Role of Mesenchymal Stem Cells in the Treatment of Idiopathic Pulmonary Fibrosis	Phase 1	The Prince Charles Hospital Mater Medical Research Institute
NCT02509156	Stem Cell Injection in Cancer Survivors	Phase 1	The University of Texas Health Science Center, Houston National Heart, Lung, and Blood Institute (NHLBI)
NCT02379442	Early Treatment of Acute Graft Versus Host Disease With Bone Marrow-Derived Mesenchymal Stem Cells and Corticosteroids	Phase 1 Phase 2	National Heart, Lung, and Blood Institute (NHLBI) National Institutes of Health Clinical Center (CC)
NCT01087996	The Percutaneous Stem Cell Injection Delivery Effects on Neomyogenesis Pilot Study (The POSEIDON-Pilot Study)	Phase 1 Phase 2	University of Miami National Heart, Lung, and Blood Institute (NHLBI) The Emmes Company, LLC
NCT02013674	The TRansendocardial Stem Cell Injection Delivery Effects on Neomyogenesis Study (The TRIDENT Study)	Phase 2	The Emmes Company, LLC University of Miami
NCT01392625	PercutaneOus StEm Cell Injection Delivery Effects On Neomyogenesis in Dilated CardioMyopathy (The POSEIDON-DCM Study)	Phase 1 Phase 2	National Heart, Lung, and Blood Institute (NHLBI) University of Miami
NCT00768066	The Transendocardial Autologous Cells (hMSC or hBMC) in Ischemic Heart Failure Trial (TAC-HFT)	Phase 1 Phase 2	University of Miami The Emmes Company, LLC
NCT00629018	Safety and Efficacy Study of Stem Cell Transplantation to Treat Dilated Cardiomyopathy	Phase 2	University Medical Centre Ljubljana Blood Transfusion Centre of Slovenia Stanford University
NCT00927784	Effect of Intramyocardial Injection of Mesenchymal Precursor Cells on Heart Function in People Receiving an LVAD	Phase 2	Icahn School of Medicine at Mount Sinai National Heart, Lung, and Blood Institute (NHLBI) Angioblast Systems

N/A, not applicable

In this review, we will focus on the major challenges of MSC therapies and the underlying factors leading to the failure of clinical trials. Recent advances and prospects concerning the translation of MSC techniques into clinical practices will also be discussed.

## Challenges in technology transfer of MSCs from bench to bedside

**Fig. 2:**
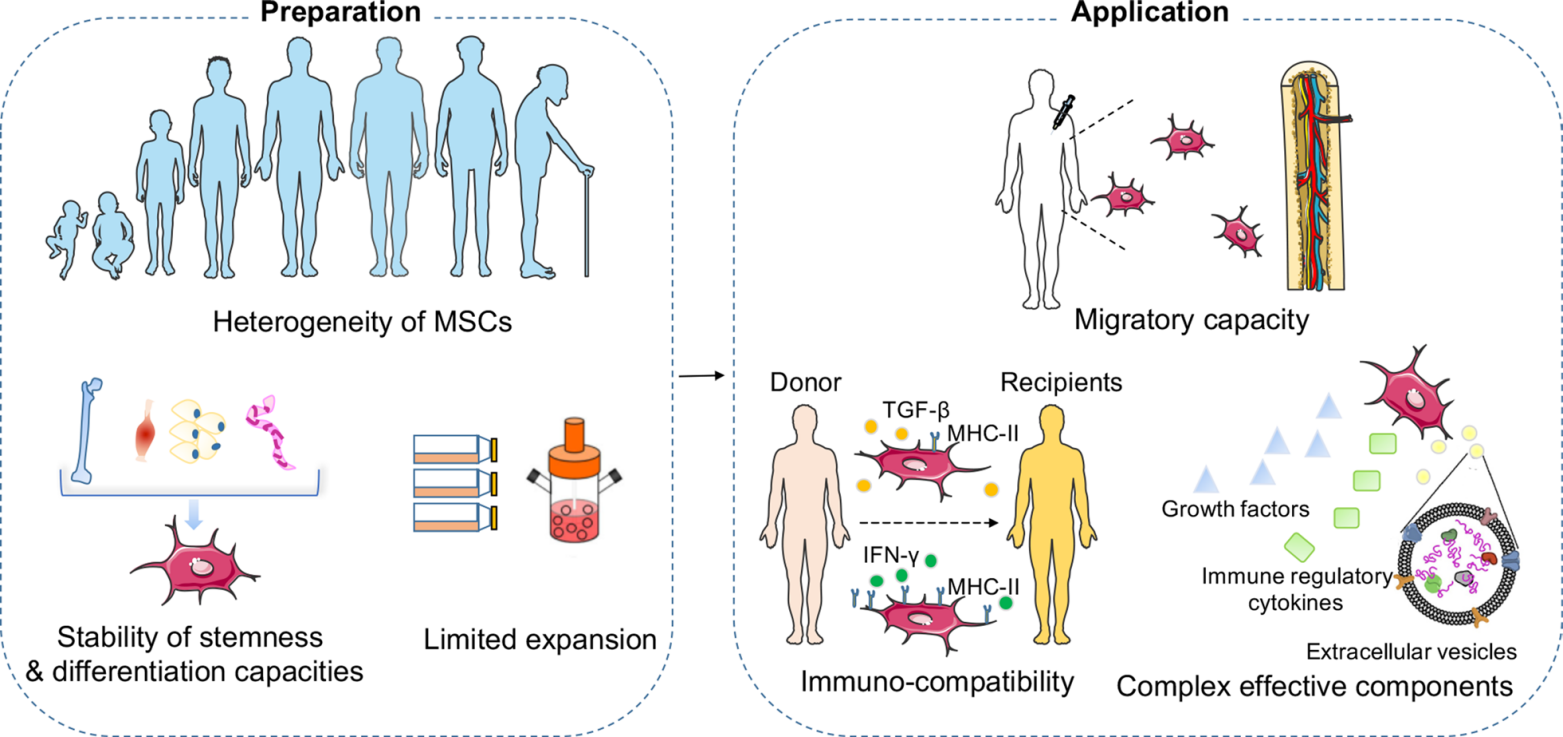
The main challenges in clinical applications of MSCs. During preparation of the MSC products, the main challenges include: (1) heterogeneity of MSCs resulted from donor variations such as the health status, genetics, gender, and age. (2) The varying degree of stability of stemness and differentiation capacities between MSCs isolated from different sources, such as bone marrow, adipose tissue, umbilical cord, or muscles. (3) The varying level of expansion capacities under different culture conditions, including confluence, culture surface, oxygen levels, flasks/bioreactors, passage number, and cell surface modifications. At the state of application, challenges remain due to the influence of (1) the homing or migratory capacity of MSCs under different administration route (local/systemic), injection site, infusion time, and cell carrier materials. (2) The immune compatibility between donors and recipients is the key to reduce the risk of rejection, but is affected by environmental inflammatory molecules which could induce distinct expression of MHC-II in MSCs. (3) The complex effective components released by MSCs depending on the host microenvironment (inflammation status, hypoxia, and ECM), which can result in highly variable factors shaping distinct functions of MSCs

Although transferring MSCs from bench to bedside is theoretically achievable, substantial failures have been reported in many early- or late-stage clinical trials, which account for the disapproval of many products by FDA [13]. Factors contributing to the failure of MSC clinical development include but not limited to the poor-quality control and inconsistent characteristics of MSCs in terms of immunocompatibility, stability, heterogeneity, differentiation, and migratory capacity [14, 15] (Fig. 2).

### Immunocompatibility of MSCs

MSCs were immune privileged due to the low expression of MHC-I and HLA-I, and no expression of HLA-II or costimulatory factors such as CD40, CD80 and CD86. MSCs can be transplanted as allogeneic cells with a low risk of rejection. Generally, the original MSCs are believed to have low immunogenicity [16]. Most MSC products are manufactured by amplifying a small number of cells obtained from donors, which can increase MSC immunogenicity caused by inappropriate processes and culture conditions. After MSCs infusion, the in vivo inflammatory molecules in turn increase MSC immunogenicity and further decrease MSCs viability and differentiation capacity, particularly when administrating xenogenic MSCs including human MSCs in animal models [17]. Although the primary immunogenicity of MSCs derived from in vitro experiments might be minimal, the secondary immunogenicity induced by in vivo positive feedback loops can cause the absence of efficacy reported in most clinical trials.

Studies have shown that inflammatory molecules (such as interferon-γ), increased cell density, and/or serum deprivation can induce high expression of MHC-II in MSCs, while TGF-β suppresses MHC-II expression [18]. The immune compatibility between donors and recipients is the key to reduce the risk of rejection in the event of long-term treatments with repeated infusions, in conditions requiring promotion of transplanted bone marrow integration, or post-renal transplantation rejection treatments [19]. It has been reported that repeated intra-articular injection of allogeneic MSCs is more likely to cause an adverse reaction than autologous cells when administered in the same manner [20]. The same observations were reported in horses treated with intracellular xenogen-contaminated autologous MSCs (such as FBS) or non-xenogen-contaminated allogeneic MSCs [21].

MSCs of high quality is the first step to ensure the safety and efficacy in clinical trials. Understanding the molecular and cellular mechanisms underlying the immune incompatibility of MSCs will help to improve the manufacture of MSC products.

### Stemness stability and differentiation of MSCs

MSCs have mesodermal lineage differentiation potential and the potential to regulate tissue regeneration by mediating tissue and organ repair, as well as replacing damaged cells [22]. Different tissue-derived MSCs exhibit tendencies to differentiate into different end-stage lineage cells [23, 24], and such regeneration and differentiation contribute to distinctive clinical efficacy.

Several laboratories have analyzed the proteome modifications associated with MSCs differentiation [25, 26]. They indicated that ‘‘stemness’’ genes were highly expressed in undifferentiated and de-differentiated MSCs [27, 28]. These highly stemness-related gene clusters in MSCs have been found to be mainly involved in the proliferation, differentiation, and migration [29]. When MSCs differentiated into osteoblasts, chondrocytes, and adipocytes, expressions of these genes significantly decreased, underlining their unique characteristics. Table 2 lists typical stemness genes of MSCs.

**Table 2:** Some typical stemness genes of MSCs

Abbreviation	Names	Functional description	References
HMGB1	High Mobility Group Box 1	Interacts with SDF-1 and CXCR4; required for tissue repairment	[30]
KLF2	Krüppel-like Factor 2	Enhances MSC proliferation; required for the maintenance of stemness	[31]
MCM2	Minichromosome maintenance marker 2	Required for cell division and DNA replication	[32]
CCNA2	Cyclin A2	Regulates cell cycle	[33]
PCNA	Proliferating cell nuclear antigen	Recruits and retains many enzymes required for DNA replication and repairment	[34]
POLA1	DNA Polymerase Alpha 1	Required for DNA replication	[35]
POLD1	DNA Polymerase Delta 1	Required for DNA replication	[36]
RFC4	replication factor C subunit 4	Required for DNA replication	[37]
MAD2L1	mitotic arrest-deficient 2 like 1	Executes mitotic checkpoint	[38]
CDK1	Cyclin-Dependent Kinase 1	A catalytic subunit of a protein kinase complex that induces cell entry into mitosis	[39]
CCNB1	Cyclin B1	Predominantly expressed in the G2/M phase of cell division	[40]
CDC45	Cell Division Cycle 45	An important component of the replication fork, in DNA unwinding	[41]
TUBA1B	Tubulin Alpha 1b	Mitosis, cell movement, intracellular movement, and other biological processes	[42]
E2F1	E2F Transcription Factor 1	Promotes proliferation or apoptosis in response to DNA damage	[43]
BIRC5	Baculoviral IAP Repeat Containing 5	Regulates apoptosis	[44]
BLM	Bloom syndrome, RecQ helicase-like	Maintains genome integrity	[45]
ITGAV	Integrin Subunit Alpha V	Belongs to α-V integrin family, required for cell surface adhesion	[46]
MAD2L1	Mitotic spindle assembly checkpoint protein MAD2A	Required for chromosomes alignment at metaphase plate	[47]

Serial passaging in long-term culture could negatively affect the expression of stemness genes [48, 49]. A previous study indicated that CD13, CD29, CD44, CD73, CD90, CD105, and CD106 in MSCs are down-regulated during culture expansion compared to MSCs in the stromal fraction [50]. The senescence-related proteins p53, p21, and p16 expressed under different conditions [51]. Rene et al. reported that after short-term in vitro culture, wild-type MSCs became senescent, and p21(−/−)p53(+/+) MSCs showed an elevated spontaneous apoptosis rate but no sign of tumoral transformation [52]. On the other hand, Mclean et al. discovered cancer-associated MSCs (CA-MSCs), which are determined by the expression of CD44, CD73, and CD90, exhibited the upregulation of the TGF-β superfamily/bone morphogenetic protein (BMP) family [53], and MSCs harbored the potential to differentiate into cancer-associated fibroblasts (CAFs) at latter passages [54–57]. The malignant phenotypes of MSCs associated with CAFs could express Meflin, which is also a marker of MSCs maintaining their undifferentiated state [57–59].

To provide sufficient MSCs for clinical trials, MSCs need to be amplified in a large scale, which will inevitably face the issue of MSCs senescence and subsequent modifications of gene expressions [60]. Therefore, the long-term culture of MSCs often results in decreased proliferation and differentiation capacities and shortened life expectancy [61]. A standardized manufacturing process is essential for the success of clinical trials. Though the above molecules have been found to mediate the stemness of MSCs and regulate their differentiation, it remains challenging to control the fate of MSCs in a complex in vivo environment.

### Heterogeneity of MSCs

**Fig. 3:**
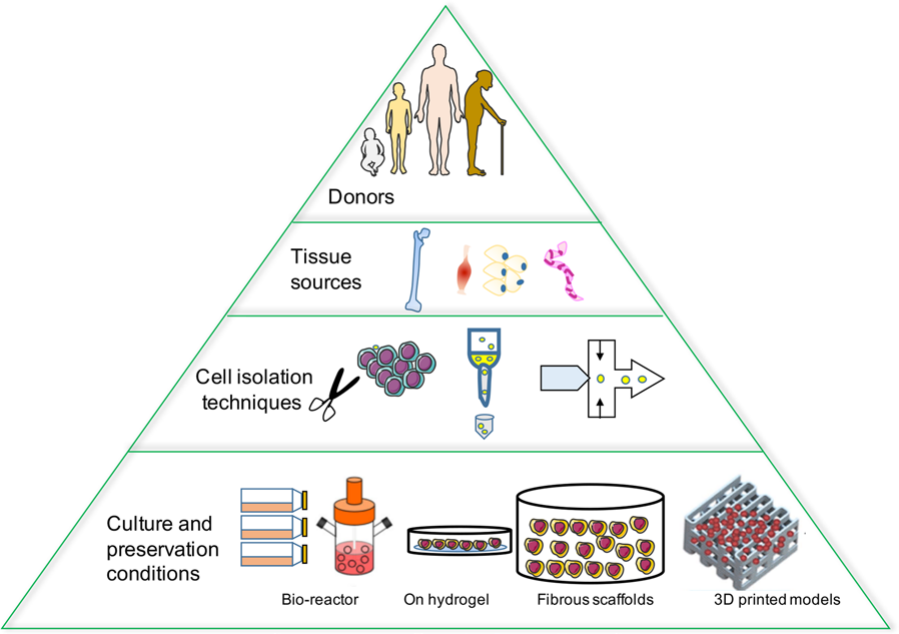
MSCs exhibit heterogeneity at multiple levels. Heterogeneity of MSCs is determined by factors at multiple levels. (1) Donors at different health status, genetics, gender, and age may result in variations. (2) Tissue from different sources exhibits distinct characteristics, therefore leading to heterogeneity. (3) Cell isolation techniques may lead to distinct purity and sub-populations. (4) Cell culture environment and preservation conditions could affect the expansion and states of MSCs, therefore also affecting the heterogeneity

Heterogeneity of MSCs is determined by multiple factors including but not limited to donors and tissue sources, cell populations, culture conditions, cell isolation techniques, cryoprotective and thawing protocols [62–64] (Fig. 3).

MSCs were defined as adherent cells with a spindle-shaped morphology in standard culture conditions according to the minimal criteria developed by the International Society of Cell Therapy in 2006 [65]. They were characterized by the following features: (1) expression of CD105, CD73, and CD90, but no expression of CD45, CD34, CD14 or CD11b, CD79a, CD19, or HLA-DR; (2) capacity to differentiate into osteoblasts, adipocytes, and chondroblasts in vitro. However, these criteria were insufficient to define MSCs as variations exist at multiple levels. First, MSCs from different donors have distinct functions due to differences in age, health condition, and other individual characteristics. Second, MSCs from different tissues ranging from adipose tissue to bone marrow could be distinct in terms of surface markers and differentiation capacities. This variation probably results from different biological, chemical, and mechanical stresses in stem cell niches, though the culture conditions are similar in vitro. Moreover, MSCs form clones, and cell heterogeneity exists both inter-clonally and intra-clonally. Extracellular matrix genes and osteogenic transcription factor-related genes show increased expression in highly osteogenic clones compared to poor osteogenic clones. Cell morphology and differentiation ability within one clone can also be remarkably different. For instance, cells located at the outer periphery express higher levels of genes related to cell proliferation (MKI67 and PODXL), while extracellular matrix genes (VCAM1) tend to be expressed in interior MSCs [66].

To identify specific cell subsets in heterogeneous MSCs, researchers have been continuously exploring characteristic cell surface markers and molecular signatures. Single cell-derived colony with rapidly dividing cells shows high colony-forming efficiency. STRO-1, CD146, and CD271 have been identified as cell surface markers for this subset [67]. However, cell subsets sharing similar surface markers would exhibit different chondrogenic differentiation capacities even under the same culture conditions [68]. RNA sequencing and microarray analysis have showed transcriptional signals predicting differentiation potential. Osterix and distal-less homeobox5 are the main transcription factors involved in osteoblast differentiation, while peroxisome proliferator-activated receptor gamma (PPAR-γ) and CCAAT/enhancer-binding protein alpha are associated with adipogenic potential [69]. In addition, MSCs with specific surface markers of differentiation potential may present various physiological functions [70]. For example, CD105 + MSCs exhibited myogenic potential assisting the repairment of the infarcted myocardium [71], while CD106 + MSCs showed enhanced multipotency and immunosuppressive ability [72]. Increasing evidence shows that MSCs comprise multiple subsets with specific surface markers. More work is needed to define these subpopulations based on biomarkers and biological functions.

### Directed migratory capacity of MSCs

The therapeutic efficacy of MSCs is highly dependent on their in vivo migration and homing capacities. The migrating direction is determined by chemokine receptors expressed on MSCs and chemokines in tissues [73]. Freshly isolated MSCs have a good homing effect, which is decreased after somatic expansion. For example, the chemokine receptor CXCR4 is highly expressed on primary bone marrow MSCs, but gradually lost with passages, resulting in the less recognition of its ligand CXCL12 (also known as SDF-1α) [74, 75]. Together, the primary MSCs are expected to have a better therapeutic efficacy due to more potent migration capacity.

However, the expression profile of chemokines in damaged tissues is often not compatible with that of receptors on MSCs. For instance, CXCL1, CXCL2 and CCL7 increased in infarcted myocardium, while expression of corresponding receptors (CCR1 and CXCR2) on MSCs was very low, resulting in low efficiency in the migration of MSCs to infarct sites [76]. To improve the migration rate, MSCs are genetically modified to express specific chemokine receptors [73]. For example, CCR7-modified MSCs efficiently migrated to secondary lymphoid organs and demonstrated significant clinical efficacy in the GVHD mouse model [77, 78]. CXCR5-modified MSCs migrated to the damaged sites by binding to CXCL13, which was highly expressed in damaged tissues [79]. Taken together, genetically modified MSCs are an independent treatment entity and could be used as targeted therapy.

The delivery of MSCs emerges as a prerequisite to the unfoldment of their full therapeutic potential. Different delivery routes could affect cell homing, survival, and paracrine function. Systemic delivery is considered a reasonable approach. However, the reported effect in terms of homing rate, survival rate, and maintenance of cellular function was modest and transient [80] for reasons including poor migration rate from vessels to tissues and high retention rate in the liver, lungs, and spleen [81]. In contrast to intravenous delivery, intra-tissue or intra-organ delivery showed higher delivery retention and efficiency, as evidenced by a large body of studies [82]. However, clustering of MSCs and occlusions in microvasculature has been reported in some disease models such as myocardial infarction [83]. Walczak et al. reported that only cells with a diameter between 20 and 50 μm could avoid intracerebral entrapment [84]. Therefore, to maximize therapeutic efficacy, both the migratory capacity of MSCs and appropriate delivery methods should be considered.

### Limited expansion of MSCs

Theoretically, MSCs can be expanded in vitro in traditional culture plates and flasks to any amount that meets experimental purpose. However, with prolonged culture duration and increased passage numbers, MSCs reach the Hayflick limit, exhibiting a marked decrease in proliferation with a transformation in morphology from a thin spindle shape to a flattened square shape. The cell density seeded in the culture containers also plays a role in the senescence of MSCs. Neuhuber et al. found the optimal cell growth of rat MSCs at 200 cells per cm^2^ compared with 20 cells or 2000 cells per cm^2^ [85]. In other studies, a relatively low density (~ 1.5–200 cells per cm^2^) was suggested to support better proliferation [86]. Alterations in autocrine secretion and contact inhibition may contribute to the slow growth at high density.

Large-scale expansion in 2D plates over long term also impacts stem cell characteristics of MSCs. According to Zhao et al., hUC-MSCs at various passages have multiple mutation spectra on signatures and functions, and cells at high passage showed declined therapeutic effect in aGVHD mouse model [87]. It has been shown that chondrogenic differentiation of MSCs in 2D culture is less efficient than that of MSCs in 3D culture [88]. Therefore, 3D expansion of MSCs was developed to prevent phenotypic changes caused by monolayers, where a broad and flattened morphology upon passaging was well preserved.

Moreover, MSCs have shown the capacity to differentiate into numerous cell types such as neural cells, hepatocyte-like cells, and pancreatic islet-like cells [89, 90]. The transient differentiation of MSCs into neural precursor-like cells may experience de-differentiation during extended culture [91]. Therefore, in vitro induction is often insufficient to yield pure functionally competent cells.

Taken together, developing the technique that can produce a huge number of cells rapidly and cost-effectively with guaranteed cell quality is paramount for the clinical progress of MSCs.

### Effective components of MSC treatments

The secretion of cytoprotective factors by MSCs was first reported by Gnecchi and colleagues. They observed that Akt-MSCs (MSCs overexpressing Akt) prevented ventricular remodeling and improved the heart function following surgical myocardial infarction (MI). Since cell transplantation and myogenic pathways would be ineffective over such a brief interval, a new mechanism was proposed that the injected MSCs might act through releasing trophic factors that contribute to myocardial protection following an ischemic insult. This hypothesis was then confirmed by evident improvements in cardiac performance following injection of conditioned medium (CM) collected from hypoxic Akt-MSCs into an induced MI model, which protected ventricular cardiomyocytes with less apoptosis when subjected to a hypoxic condition [92].

In 2007, Dai et al. observed that MSCs-CM had a similar, albeit less intense, effect of MSCs in myocardial infarction, indicating that at least part of the effect observed following MSCs injection could be attributed to soluble factors [93]. In the context of neuronal damage, it has been established that the presence of BDNF, GDNF, NGF, and IGF in the MSCs secretome is necessary for the neuronal survival in vitro and in vivo [94, 95]. MSCs-CM has demonstrated therapeutic efficacy in some other disease models including chronic kidney disease, certain lung, and liver diseases [96, 97].

The paracrine effects of MSCs as an initial mechanism of action inspired further biological analysis of MSCs secretome [98]. Subsequent studies found more paracrine effectors, including soluble cytokines, growth factors, hormones, miRNAs, or lncRNAs that targeting a variety of cells such as immune cells and injured tissue cells [99]. In addition, the paracrine effectors could be loaded in extracellular vesicles (EVs) and exerted long-term effects [100]. In accordance, many studies have shown that MSC-derived EVs retain the biological activity of parental MSCs. It has been demonstrated that EVs showed a similar therapeutic effect as MSCs in selected animal models [101]. However, different studies found various effective components of MSCs in specific animal models and human diseases, and the interactions and functional differences between effectors remain elusive. Therefore, novel in-depth analytical techniques and platforms are warranted to investigate the MSCs secretome in the future.

## Attempts to improve the therapeutic outcomes of MSCs

**Fig. 4:**
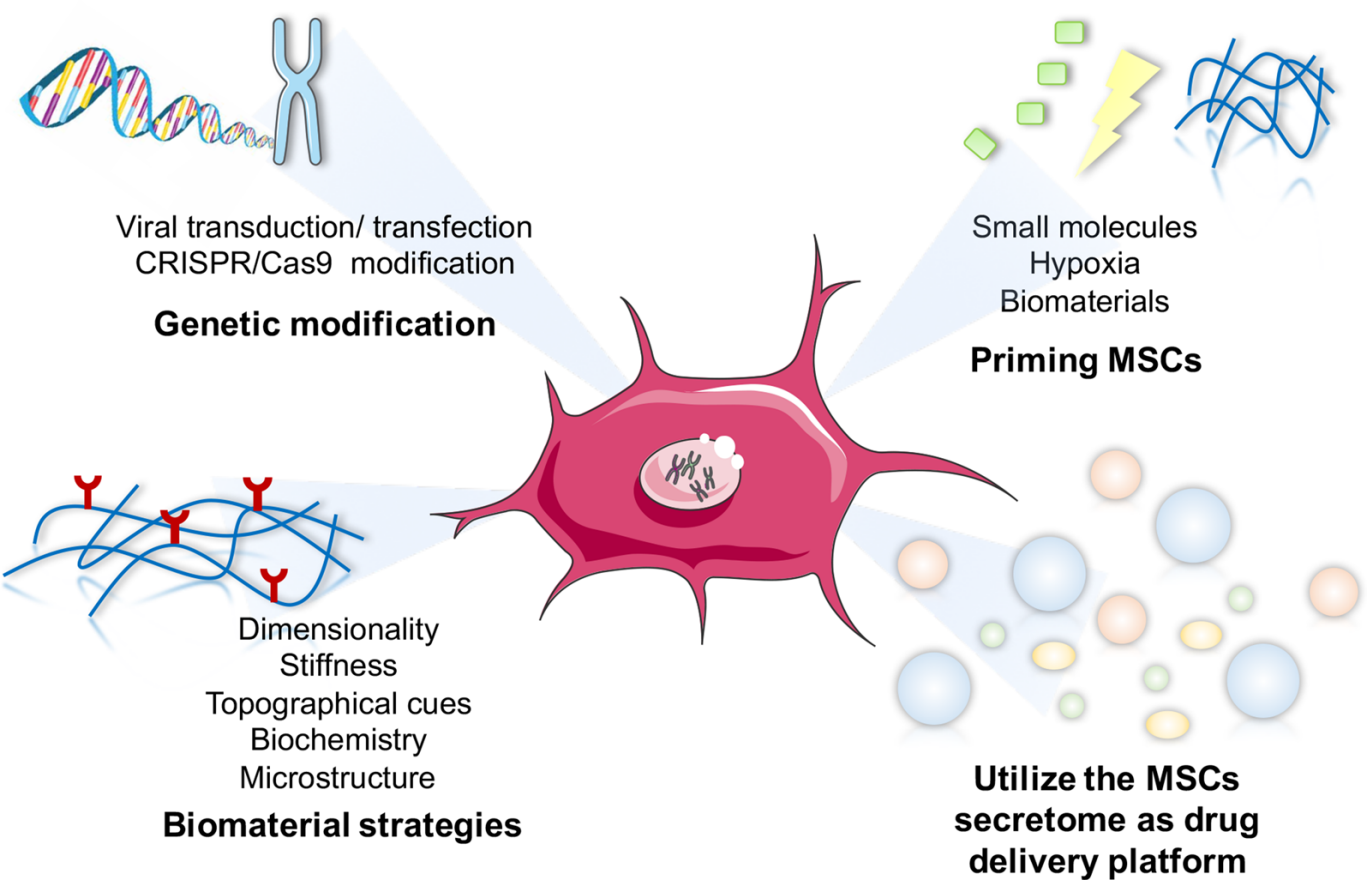
Current attempts to improve MSC treatment. To improve the therapeutic efficiency of MSCs treatment, modification was made mainly in the following aspects: (1) genetic modification of MSCs by viral transduction or CRISPR/Cas9 techniques to engineer MSCs with enhanced homing, potency, or expansion capacities; (2) priming MSCs with small molecules, hypoxia, or structural stimulations by biomaterials to improve MSC function, survival, and therapeutic efficacy, thus boosting their therapeutic efficacy; (3) biomaterial strategies to improve the survival and function of MSCs by offering a scaffold for MSCs adherence, including modifications on dimensionality, stiffness, topographical cues, surface chemistry, and microstructure of biomaterials. (4) Utilize the MSCs secretome as a drug delivery platform for treatment

Although there were no attributable serious adverse events after MSC therapy, fever within 24 h and temporary pain at the injection sites are commonly occurred. Here we summarize four strategies to limit adverse events related to MSC treatments and improve the therapeutic outcomes, including genetic modifications or priming strategies to change the inherent characteristics of MSCs, and biomaterial strategies to modify the outside circumstances, and the usage of MSCs secretome (Fig. 4).

### Biomaterial strategies to maintain more homogeneous MSCs

Biomaterials for delivering MSCs have been extensively investigated. These materials showed advantages in offering a scaffold for the adherence and survival of MSCs, as well as preserving the functional components MSCs secreted, thus elongating the effective durations in clinical treatment. However, the implantation of biomaterials could induce the foreign-body responses (FBR) in the host immune system, which can potentially result in fibrosis and failure of the implantation. Therefore, biomaterials suitable for MSCs were constructed to ameliorate the FBR and subsequent fibrotic encapsulation [102]. For example, loading MSCs with small-molecule encapsulating microparticles (MPs) can boost the duration of the products. MPs are composed of biocompatible materials that can be therapeutically tuned according to their composition, polymer molecular weight, drug loading, and release capacities [103]. MSCs loaded with degradable budesonide-containing MPs exhibited fourfold increase in IDO activity in vitro compared to MSCs without being pre-treated with budesonide [104]. This led to a twofold improvement in the suppression of peripheral blood mononuclear cells (PBMCs) activation following IFN-γ stimulation [105].

MSCs are typically delivered to a graft site using a decellularized extracellular matrix (ECM) scaffold. The advent of synthetic polymers has revolutionized tissue engineering. These polymers are highly tunable, homogenous, and cell-free materials and have a high batch-to-batch consistency taking the form of porous hydrogels, sponges, plates, or membranes [106, 107]. However, their unique properties could exert different influences on MSCs function. Table 3 summarizes the influence of biomaterials properties on the function of MSCs, including dimensionality, stiffness, topographical cues, surface chemistry, and microstructure of biomaterials.

**Table 3:** The properties of biomaterials affecting the function of MSCs

Biomaterial properties	Biomaterial	MSCs Source	Experiment model	MSCs function	References
Dimensionality	3D alginate micro-encapsulation versus 2D TCP	Human bone marrow	In vitro coculture with rat hippocampal slice	Reduced TNF-α and enhanced PGE-2 in the coculture slice	[108]
	3D alginate hydrogel versus 2D TCP	Human adipose tissue	In vitro	Enhanced potential of suppressing the proliferation of PBMCs	[109]
	HA hydrogel encapsulation versus free cells	Rat bone marrow	In vivo implantation into rat SCI model	Encapsulated MSCs reduced M1 macrophages	[110]
Stiffness	Fibrin hydrogel	Human bone marrow	In vitro	Changed elastic modulus of hydrogel and protein secretion levels of VEGF and PGE-2 by MSCs	[111]
	Electrospun PCL fibrous scaffolds with random, aligned, and mesh-like fiber alignment	Rat adipose tissue	In vitro	MSCs on mesh-like fibers had the greatest potential of immunomodulation	[112]
Topographical cues: fiber alignment	Electrospun PLLA fibrous scaffolds with random or aligned fiber alignment	Human adipose tissue	In vitro	MSCs on aligned fibers had enhanced expression and secretion level of TSG-6 and COX-2	[113]
	Ti-alloy disks with macro–micro-nanoscale-roughened surface or smooth surface	Human	In vitro	MSCs on rough surface had reduced secretion levels of proinflammatory gene expression	[114]
Topographical cues: surface roughness	Biphasic calcium phosphate bioceramics with micro-nanoscale-roughened surface or smooth surface	Mouse bone marrow	In vitro	MSCs on rough surface had reduced expression levels of proinflammatory cytokines	[115]
Topographical cues: surface structure	Thermoplastic polyurethane plates with grid-like cavities or no-structure	Human bone marrow	In vitro	MSCs on grid-like structure had enhanced secretion levels of PGE-2 and IL-1RA	[116]
Biochemistry	HA with different molecular weights: 1.6 MDa, 150 kDa, or 7.5 kDa	Human bone marrow	In vitro	Molecular weight of HA had negligible effect on MSC expression levels of immune modulators	[117]
Micro-structure	Type-I collagen hydrogel, sponge and membrane	Neonatal rabbit bone marrow	In vitro	MSCs in a hydrogel that has the smallest pore size showed the greatest suppressive effect on the proliferation of PBMCs	[118]

### Genetic modification to produce MSCs with desired biologic function

#### Viral DNA transduction and mRNA/DNA transfection

To further optimize the therapeutic efficacy of MSCs, MSCs have been genetically engineered to produce trophic cytokines or other beneficial gene products in numerous preclinical models by transfecting MSCs with viral or non-viral vectors. Over the last few decades, these MSCs have successfully been engineered to express therapeutic peptides and proteins in animal models [119]. For instance, MSCs expressing thioredoxin-1 (Trx1, a powerful antioxidant, transcription factor and growth factor regulator) improved cardiac function in post-myocardial infarction rat models [120]. MSCs expressing IL-12 showed potent anticancer activity against melanoma, breast cancer, and hepatoma [121, 122]. And MSCs expressing interferon-γ inhibited tumor growth in mouse neuroblastoma and lung carcinoma models [123, 124]. In line with these advances achieved in animal models, several MSCs-based therapies are under clinical development (Table 4).

**Table 4:** Engineered MSCs for treatment reaching the clinical stage

Delivery system	Administration route	Sponsor	Indication	Development phase	Status	NCT number
MSCs secreting IFN-*β*	Intraperitoneal	M.D. Anderson Cancer Center, Dallas TX	Ovarian cancer	Phase 1	Active, not recruiting	NCT02530047
MV-NIS infected adipose tissue–derived MSCs	Intraperitoneal	Mayo Clinic, Rochester MN	Recurrent ovarian cancer	Phase 1/2	Recruiting	NCT02068794
Bone marrow-derived autologous MSCs infected with ICOVIR5, an oncolytic adenovirus (CELYVIR)	Intravenous	Hospital Infantil Universitario Niño Jesús, Madrid, Spain	Metastatic and refractory solid tumors	Phase 1/2	Completed	NCT01844661
MSCs genetically modified to express TRAIL	Intravenous	University College, London	Lung adenocarcinoma	Phase 1/2	Recruiting	NCT03298763
Autologous human MSCs genetically modified to express HSV-TK	Intravenous	Apceth GmbH & Co. KG, Germany	Advanced gastrointestinal cancer	Phase 1/2	Completed	2012–003,741-15 (EudraCT number)

However, both viral and non-viral vectors have some limitations. Non-viral vectors present transient gene expression and low-transfection efficiency, while viral transduction is associated with a higher risk of chromosomal instability, insertional mutagenesis, and proto-oncogene activation despite the inherent high transfection efficiency [125]. The adverse immune reactions induced by viral transduction were reported to impair the stability of transgenes [126, 127]. Therefore, the limitations and adverse responses should be valued when modifying MSCs by transfection.

Some studies made attempts on human-induced pluripotent stem cell (iPSC)-derived MSCs to obtain improved expandability. Actually, therapeutic transgenes could be inserted into iPSC-derived MSCs before MSCs derivation. This strategy could eliminate insertional mutation as well as guarantee stable expression of transgenes during prolonged expansion [128]. So iPSC-derived MSCs may be a candidate of MSCs for usage.

#### CRISPR-Cas9 technology to obtain highly homogeneous MSCs

With CRISPR/Cas9 technology, genetic modification of MSCs can be done with higher efficiency and specificity [129]. Compared to transcription activator like effector nuclease (TALEN) and the zinc-finger nucleases (ZFNs), CRISPR/Cas9 technology is faster, more economically efficient, and user-friendly [130]. CRISPR/Cas9-based gene manipulation has been widely employed in stem cell field particularly MSCs research, including gene knock-in, knock-out, activation or silence, etc.

CRISPR/Cas9-mediated gene knockdown in MSCs has been proved effective in treating diseases such as myocardial infarction [131]. Targeted gene knock-in promoted the differentiation capacity of MSCs and, in turn, ameliorated the insufficiency of functional cells in local sites [132]. Genetically modified MSCs have been evaluated in clinical trials. The TREAT-ME-1 study, an open-label, multicenter, and first-in-human Phase 1/2 trial, evaluated the safety, tolerability, and efficacy of genetically modified autologous MSC-apceth-101 treatment in patients with advanced gastrointestinal adenocarcinoma [133]. Further investigations are still needed to obtain unequivocal evidence on the differentiation and regeneration potentials of MSCs in vivo. Moreover, next-generation sequencing and genotypic techniques might serve as a new paradigm to improve the efficacy on targeting specific cell types for personalized medicine. CRISPR gene-engineered MSCs studies are illustrated in Table 5.

**Table 5:** The tests of modified MSCs using CRISPR-Cas9 technology

Source of MSCs	Gene	Outcome	References
Human umbilical cord-derived MSCs	MCP-1/CCL2	CCL2-overexpressing hUC-MSCs showed better functional recovery relative to naïve hUC-MSCs, promoting subsequent endogenous brain repair	[134]
Human pancreatic ductal tissue MSCs	PTEN gene	PTEN mRNA synthesized in vitro is capable of being applied to a MSC-mediated anticancer strategy for the treatment of glioblastoma patients	[135]
Mouse bone marrow MSCs	SV40T into a safe harboring site at Rosa26 locus	CRISPR/Cas9 HDR-mediated immortalization of BMSCs can be more effectively reversed than that of retrovirus-mediated random integrations	[136]
Human bone marrow MSCs	Promotor of ectodysplasin (EDA)	After transfection with sgRNA-guided dCas9-E, the BM-MSCs acquired significantly higher transcription and expression of EDA by doxycycline (Dox) induction	[137]
Mouse bone marrow-derived MSCs	IL-10	Transplantation of CRISPR system engineered IL10-overexpressing bone marrow-derived MSCs for the treatment of myocardial infarction in diabetic mice	[138]
Rat bone marrow MSCs	Smad7	Smad7-MSCs is effective in treating liver fibrosis in the CCl4-induced liver cirrhosis model via inhibition of TGF-β1 signaling pathway	[139]
Human mesenchymal stem cells	First intron of the PPP1R12C gene	exogenous gene hFIX was effectively expressed following site‑specific targeting into the AAVS1 locus in MSCs; MSCs may be used as potential cell carriers for gene therapy of hemophilia B	[140]
Immortalized human bone marrow MSC cell line (ATCC PCS-500–041)	PUMILIO2 (PUM2)	Depletion of PUM2 blocks MSC adipogenesis and enhances osteogenesis. PUM2 works as a negative regulator on the 3′ UTRs of JAK2 and RUNX2 via direct binding. CRISPR/CAS9-mediated gene silencing of Pum2 inhibited lipid accumulation and excessive bone formation	[141]
Human bone marrow-MSCs	Platelet-derived growth factor B (PDGF-B)	PDGFB-MSCs increased anti-apoptotic signaling and exhibited enhanced survival and expansion after transplantation, resulting in an enlarged humanized niche cell pool that provide a better humanized microenvironment to facilitate superior engraftment and proliferation of human hematopoietic cells	[142]

Despite the specificity of CRISPR/Cas technology in gene delivery [143], only one clinical trial of MSCs modified with CRISPR/Cas9 has been registered (NCT03855631).

### “Priming” MSCs with small molecules to exogenously boost their therapeutic function

Given current manufacture of MSCs cannot meet the requirement for clinical trials in terms of production scale, the alternative is to boost the function of limited cells through priming MSCs. Priming has also been referred to as licensing or preconditioning, which is a concept commonly used in the field of immunology, and it has been adapted to the scope of stem cells [144, 145]. One of the commonly used strategies is priming MSCs with pro-inflammatory mediators, including IFN-γ, TNF-α, IL-1α, and IL-1β, and more priming approaches are being proposed to improve the function, survival, and therapeutic efficacy of MSCs [146, 147]. The priming approaches could be divided into three categories based on the stimulations: (a) MSCs priming with small molecules, (b) MSCs priming with hypoxia, (c) MSCs priming with biomaterials. Table 6 summarizes some representative priming MSCs.

**Table 6:** Representative priming MSCs in preclinical and clinical studies

Stimuli	Source MSCs	Model/disease	In vivo*/*in vitro	Results	References
IFN-γ	Bone marrow	graft-versus-host disease (GVHD)	In vivo	IFN-γ primed MSCs significantly reduced the symptoms of GVHD in NOD-SCID mice, thereby increasing survival rate when compared with naïve MSC-infused mice	[146]
IFN-γ	Bone marrow	–	In vitro	Inhibited T cell effector function through the ligands for PD1 and Th1 cytokines production	[148]
IFN-γ	Bone marrow	IDO1, which depletes tryptophan necessary to support proliferation of activated T cells	In vitro	MSCs priming causes chromatin remodeling at the IDO1 promoter, that this alteration is maintained during processing commonly used to prepare MSCs for clinical use and that, once primed, MSCs are poised for IDO1 expression even in the absence of cytokines	[149]
IFN-γ	Bone marrow	–	In vitro	Xenotransplantation of IFN-γ-pretreated human MSCs induces mouse calvarial bone regeneration	[150]
IFN-γ	Bone marrow	DSS-induced colitis model	In vitro*/* in vivo (mice)	Attenuated development of colitis, reduced pro-inflammatory cytokine levels in colon and increased migration potential	[151]
IFN-γ	Umbilical cord	–	In vitro	Increased suppression of NK cells and reduced NK-mediated cytotoxicity	[152]
IL-1β	Umbilical cord	DSS-induced colitis model	In vitro*/* in vivo (mice)	Attenuated the development of murine colitis, increased migration potential to inflammatory sites by CXCR4 upregulation	[153]
TNF-α and LPS	Bone marrow	–	In vitro	Increased alkaline phosphate activity and bone mineralization	[154]
IL-17A	Bone marrow	–	In vitro	Increased suppressive potential of T cell proliferation correlated with increased IL-6, inhibited surface CD25 and Th1 cytokines expression, and induced iTregs	[155]
5% O2	Wharton’s jelly	–	In vitro	Conditioned-medium increased migration and tube formation in vitro, partially reduced by prior inhibition autophagy	[156]
2.5% O2	Bone marrow	Radiation-induced lung injury model	In vitro*/* in vivo (mice)	Upregulated HIF-1α, increased survival and the antioxidant ability, increased efficiency in the treatment of radiation-induced lung injury	[157]
2–2.5% O2	Placenta	–	In vitro	Upregulated glucose transporters, adhesion molecules and increased angiogenic potential	[156]
2% O2	Adipose tissue	Murine hindlimb ischemia model	In vitro*/* in vivo (mice)	Enhanced proliferation, survival, and angiogenic cytokine secretion in vivo	[158]
1.5% O2	Bone marrow	Bleomycin-induced pulmonary fibrosis model	In vitro*/* in vivo (mice)	Improved pulmonary functions and reduced inflammatory and fibrotic mediators in vivo	[159]
1% O2	Human cord blood	–	In vitro	Increased the survival and pro-angiogenic capacity in ischemia-like environment, induced anti-apoptotic mechanisms, and increased VEGF secretion	[160]
1% O2	Bone marrow	Intramuscular injection into immune-deficient mice	In vitro*/* in vivo (mice)	Reduced cell death under serum-deprivation conditions, decreased cytochrome c and HO-1 levels, enhanced survival in vivo	[161]
3D cell culture in collagen-hydrogel scaffold	Umbilical Cord	–	In vitro	Induced chondrogenesis differentiation by increasing expressions of collagen II, aggrecan, COMPS	[162]
3D cell culture in chitosan scaffold	Bone marrow (rat)	–	In vitro	Induced chondrogenesis differentiation by increased production of collagen type II	[163]
3D cell culture of composite combining an affinity peptide sequence (E7) and hydrogel	Bone marrow (rat)	–	In vitro	Increased cell survival, matrix production, and improved chondrogenic differentiation ability	[164]
3D cell culture in hydrogel	bone marrow (Human)	Rat myocardial infarction model	In vitro*/* in vivo	The epicardial placement of MSC-loaded POx hydrogels promoted the recovery of cardiac function and structure with reduced interstitial fibrosis and improved neovascular formation	[165]
Encapsulation in hydrogel	Bone marrow (rat)	Diabetic ulcers model	In vitro*/* in vivo (rats)	Promoted granulation tissue formation, angiogenesis, extracellular matrix secretion, wound contraction, and re-epithelialization	[166]
High glucose concentration in the culture medium	Bone marrow		In vitro	Decreased chondrogenic capacity	[167]
Medium from cardiomyocytes exposed to oxidative stress and high glucose	Bone marrow (diabetic mouse)	Diabetes induced with streptozotocin model	In vitro*/* in vivo (mice)	Enhanced survival, proliferation and angiogenic ability, increased the ability to improve function in a diabetic heart	[168]
Spheroid formation (different techniques)	Bone marrow		In vitro	Enhanced homogenous cellular aggregates formation and improved osteogenic differentiation (low attachment plates)	[169]
Spheroids formation (hanging-drop)	Bone marrow	Zymosan-induced peritonitis model	In vitro*/* in vivo (mice)	Expressed high levels of anti-inflammatory (TSG-6 and STC-1) and anti-tumorigenic molecules compared to 2D culture, suppressed inflammation in vivo	[170]
matrilin-3-primed spheroid generation	Adipose tissue	intervertebral disc (IVD) degeneration	In vitro*/* in vivo (rabbit)	Priming MSCs with matrilin-3 and spheroid formation could be an effective strategy to overcome the challenges associated with the use of MSCs for the treatment of IVD degeneration	[171]
Spheroids formation (hanging drop)	Cord blood	Hindlimb ischemia model	In vitro*/* in vivo (mice)	Improved engraftment; increased the number of microvessels and smooth muscle α-actin-positive vessels	[172]

“Priming” MSCs resulted in exogenously boosted therapeutic function in comparison with original state. Several “primed” MSC products have been applied clinically, with the most notable being NurOwn from Brainstorm Cell Therapeutics Company. NurOwn boosted the expression of multiple neurotrophic factors (NTFs) including GDNF, BDNF, VEGF, and HGF [173]. When administered to patients with neurodegenerative diseases, NurOwn delivered multiple NTFs as well as the immunomodulatory components secreted by MSCs. This combination demonstrated impressive therapeutic efficacy in a phase 2 clinical trial (NCT02017912), in which ALS patients got reduced ALS progression 24 months after NurOwn infusion compared to the controls [174]. So the indication of NurOwn has been expanded to include multiple sclerosis.

However, priming approaches of MSCs still have many limitations in clinical translation, such as induction of immunogenicity, high costs, variable effects, and lack of good manufacturing practices (GMP) suitable for clinical application [175]. Moreover, the long-term effect of priming MSCs has not been evaluated yet. Further studies are needed to evaluate (1) the effects of different priming approaches in clinic; (2) the best sources for MSCs isolation; (3) the epigenetic modifications, immunogenicity, and tumorigenicity of primed and non-primed MSCs; and (4) the appropriate GMP standards for quality control of MSC products, including quality of cryopreserved primed-MSCs at different passages.

### Utilize the MSCs secretome as a drug delivery platform for treatment

The “secretome” of MSCs, including secretory proteins such as growth factors, cytokines, and chemokines and EVs such as microvesicles (MVs; 100–1000 nm diameter) and exosomes (40–150 nm diameter), has been shown to exhibit many of the therapeutic properties of MSCs. For example, MSC-derived EVs have demonstrated similar or even superior therapeutic capacity for autoimmune diseases and neurodegenerative disorders compared with their parental MSCs [176, 177]. They also have better safety profiles due to their better immunocompatibility. In addition, they can bypass the endothelial layers in the blood–brain barrier or blood-retinal barrier, providing an ideal cargo to deliver biomolecules to the central nervous system [178].

Several studies have demonstrated the clinical effectiveness of MSC-EVs. For example, hBMMSC-EVs revealed significant improvements in patients suffering from refractory graft-versus-host disease [179]. In another study, administration of hUCMSC-EVs resulted in overall improvement in patients with grade III-IV chronic kidney disease [180]. Nassar et al. conducted a clinical trial to assess the effects of hUCMSC-EVs on pancreatic islet beta cell mass in Type-1 diabetic patients (NCT02138331). And there are other ongoing trials conducted to determine the safety and efficacy of human MSC-EVs in ocular diseases such as promoting the healing of large and refractory macular holes (NCT03437759) and relieving dry eye symptoms in oGVHD patients (NCT04213248). Moreover, MSC-EVs have been modified to load small molecules. For example, miR-124 was loaded in exosomes to treat patients with acute ischemic stroke (NCT03384433).

## Advances and perspectives to overcome challenges in MSC clinical application

### Artificial intelligence (AI) in MSC treatment

Digital technology and AI are driving the revolution of healthcare industry [181]. The drug research and development became an important application field of AI technology [182]. AI in de novo design has successfully produced biologically active molecules with desired properties [183]. The discovery of drug molecules by AI has been selected as one of the "top ten global breakthrough technologies" by MIT Technology Review in 2020. The advances of AI are likewise expected to boost the understanding of MSCs therapies and help identifying the essential elements of MSCs.

AI can find new molecular compounds and emerging drug targets much faster than traditional methods, thus speeding up the progress of drug development [184, 185]. At the same time, AI can more accurately predict the follow-up experimental results of new drugs, so as to improve the accuracy at each stage of drug development [186]. Computer-aided drug design techniques are thus revolutionizing MSCs therapies.

To understand the essential elements in MSCs treatment, AI may recognize the dynamic molecular characteristics of essential elements, which include different protein sequences, molecular structures, as well as the binding forces and stabilities between targeted molecules and cell receptors. These data could be used to train a predictive model to the utmost accuracy [187]. Predicted elements may also be produced under AI guidance. Powered by a robotic platform, a system developed by MIT researchers partially automates the production of small molecules that could be used in medicine, solar energy, and polymer chemistry. Reportedly, the new system combines three main steps. First, software guided by AI proposes a route for synthesizing a molecule, then chemical experts review this route and refine it into a chemical "recipe," and lastly, the recipe is sent to a robotic platform that automatically assembles the hardware and performs the reactions that build the molecule [188].

At present, the pharmaceutical world is increasingly engaged in technologies to shorten the time required to identify new drugs and repurpose current drugs. Since MSC therapies showed beneficial effects with complex undetermined components, AI may be well-suited to analyzing and revealing essential elements. Companies such as Merck, GSK, and Roche have developed partnerships with AI companies to construct suitable platforms [189, 190]. However, the drug discovery process with AI is a long shot, which need to be verified in clinical trials.

### Engineered MSC-EVs for treatment

Paracrine effect was discovered to mediate MSCs therapeutic efficacy in previous studies [191–193]. EVs are one of the major paracrine effectors, which are bilayer membrane structures transferring bioactive components [194]. The best-studied EVs can be classified into exosomes and microvesicles according to their sizes, shapes, biogenesis, origins, and compositions [195, 196]. Due to their liposome-like structures reflecting biophysical characteristics of the parental cells, EVs are stable in vivo compared to other foreign particles [197]. Moreover, it is relatively easy to modify and/or improve the contents of EVs and their surface properties to enhance the therapeutic potential or to act as a drug delivery system [198]. These advantages make EVs promising for clinical treatment. Currently, there are 15 clinical trials registered in ClinicalTrial.gov (Table 7). However, none has been completed and challenges remained for the practical application of EVs.

**Table 7:** The registered clinical trials of treatment using EVs or exosomes derived from MSCs

NCT number	Title	Status	Condition	Phase	Start date
NCT04173650	MSC EVs in Dystrophic Epidermolysis Bullosa	Not yet recruiting	Dystrophic Epidermolysis Bullosa	Phase 1 Phase 2	Sep-2020
NCT04276987	A Pilot Clinical Study on Inhalation of Mesenchymal Stem Cells Exosomes Treating Severe Novel Coronavirus Pneumonia	Completed	Coronavirus	Phase 1	Feb-2020
NCT02138331	Effect of Microvesicles and Exosomes Therapy on cell Mass in Type I Diabetes Mellitus (T1DM)	Unknown status	Diabetes Mellitus Type 1	Phase 2 Phase 3	Apr-2014
NCT04313647	A Tolerance Clinical Study on Aerosol Inhalation of Mesenchymal Stem Cells Exosomes In Healthy Volunteers	Recruiting	Healthy	Phase 1	Mar-2020
NCT03384433	Allogenic Mesenchymal Stem Cell-Derived Exosome in Patients With Acute Ischemic Stroke	Recruiting	Cerebrovascular Disorders	Phase 1 Phase 2	Apr-2019
NCT04223622	Effects of ASC Secretome on Human Osteochondral Explants	Not yet recruiting	Osteoarthritis	-	Feb-2020
NCT04213248	Effect of UMSCs-Derived Exosomes on Dry Eye in Patients With cGVHD	Recruiting	Dry Eye	Phase 1 Phase 2	Feb-2020
NCT03437759	MSC-Exos Promote Healing of MHs	Recruiting	Macular Holes	Early Phase 1	Mar-2017
NCT04356300	Exosome of Mesenchymal Stem Cells for Multiple Organ Dysfunction Syndrome After Surgical Repair of Acute Type A Aortic Dissection	Not yet recruiting	Multiple Organ Failure	Not Applicable	Sep-2020
NCT04388982	The Safety and the Efficacy Evaluation of Allogenic Adipose MSC-Exos in Patients With Alzheimer's Disease	Recruiting	Alzheimer Disease	Phase 1 Phase 2	Jul-2020
NCT03608631	Exosomes in Treating Participants with Metastatic Pancreas Cancer with KrasG12D Mutation	Not yet recruiting	Metastatic Pancreatic Adenocarcinoma|Pancreatic Ductal Adenocarcinoma|Stage IV Pancreatic Cancer	Phase 1	Mar-2020
NCT04602442	Safety and Efficiency of Method of Exosome Inhalation in COVID-19 Associated Pneumonia	Enrolling by invitation	Covid19	Phase 2	Oct-2020
NCT04491240	Evaluation of Safety and Efficiency of Method of Exosome Inhalation in SARS-CoV-2 Associated Pneumonia	Completed	Covid19	Phase 1 Phase 2	July 2020
NCT04602104	A Clinical Study of Mesenchymal Stem Cell Exosomes Nebulizer for the Treatment of ARDS	Not yet recruiting	Acute Respiratory Distress Syndrome	Phase 1 Phase 2	Oct-2020
NCT03857841	A Safety Study of IV Stem Cell-derived Extracellular Vesicles (UNEX-42) in Preterm Neonates at High Risk for BPD	Recruiting	Bronchopulmonary Dysplasia	Phase 1	June-2019

First of all, the manufacture of large scales of MSC-EVs with high purity is difficult. MSC-EVs are isolated from MSC culture media, of which conditions including the seeding cell number, media volume, and isolation method and time of EVs can influence both the quantity and quality of EVs [199]. Therefore, optimization of culture methods (e.g., hypoxia, sheer stress, and bioreactor) combining with intensive evaluation of the pros and cons of the different EVs isolation methods is prerequisites for MSC-EVs to yield improvements. These procedures should be regulated and controlled to ensure the clinical-grade EVs production [200]. Recently, Mendt et al. reported using a bioreactor system in the GMP facility to obtain sterile, clinical-grade EVs from BM-MSCs. In that instance, the therapeutic effects of BM-MSCs on pancreatic cancer xenograft mouse models were evaluated, and feasible directions for clinical application of MSC-EVs were provided [201].

Safety and efficacy of MSC-EVs in various disease conditions need to be ensured in further preclinical and clinical evaluation. In vivo distribution analysis of fluorescence-labeled EVs has shown that MSC-EVs might have homing capacity for injured or tumor-bearing sites comparable as MSCs [202]. Long-term toxicity and immunogenicity of repetitive EVs administration using hematological examination, histopathological analysis, and immunotyping test should also be performed to find whether MSC-EVs might trigger immune responses or toxic reactions [203].

After the disclosure of precise mechanisms of action or key therapeutic factors in MSC-EVs therapy, targeted-EVs could be expanded in uniform proliferative cells such as fibroblasts via gene modification technology. Therefore, with big data-based analysis of transcriptome and proteome, engineered EVs may be manufactured with desired elements. For instance, Thomas C. Roberts et al. engineered EVs to express IL6 signal transducer (IL6ST) decoy receptors to selectively inhibit the IL6 trans-signaling pathway. Treatment in the Duchenne muscular dystrophy mouse model with these IL6ST decoy receptor EVs resulted in a reduced phosphorylation of STAT3 in muscles; further functional studies verified the in vivo activity of the decoy receptor EVs as a potential therapy [204]. Similarly, CXCR4/TRAIL-enriched exosomes were successfully obtained from MSCs overexpressing both CXCR4 and TRAIL. These exosomes exerted activity as a cooperative agent with carboplatin against brain metastasis of breast cancer in vivo, improving the efficacy of chemotherapy and highlighting a novel synergistic protocol with anticancer agents to treat brain diseases [205, 206]. Moreover, in a Phase 1 clinical trial, IL-12 was engineered to express on the exosome surface using Codiak’s proprietary engEx Platform. This product could enhance the dose control of IL-12 and limit systemic exposure and associated toxicity. EVs can overcome the reported limitations of parental cells on various aspects, including safety, reproducibility, and cost-effectiveness related to storage and maintenance. Engineered EVs might be novel promising therapeutics for clinical application. Furthermore, to resolve current hurdles in EVs-based therapeutics, the production of EVs should be standardized and optimized, and its underlying mechanisms need further investigation.

### MSC usage for pandemic diseases such as COVID-19

Pandemic diseases like 2019 novel coronavirus disease (COVID-19) have dramatically increased the number of sickness and death worldwide. Though vaccines have been developed recently, the viruses are still rapidly mutating and expanding, and the available specific and effective treatment options are currently very limited [207]. For severe or critical COVID-19 patients requiring hospitalization, acute lung injures (ALI)/acute respiratory distress syndrome (ARDS) was the main pathologic features, characterized by immunopathological complications with cellular fibromyxoid exudates, extensive pulmonary inflammation, pulmonary edema, and hyaline membrane formation [208]. Besides, inflammation and sepsis are also the leading causes of mortality in COVID-19 patients [209]. In all these cases, any treatment that could hasten recovery would be in substantial demand. MSC therapy may be one such treatment.

MSC therapeutics may be the ideal candidates for handling the broad spectrum of COVID-19 symptoms due to their multifactorial mode-of-action [210]. They can release various factors including keratinocyte growth factor, prostaglandin E2, granulocyte–macrophage colony-stimulating factor (GM-CSF), IL-6, and IL-13 to facilitate the phagocytosis and alternative activation of alveolar macrophages, alter the cytokine secretion profile of dendritic cell subsets, and decrease the release of interferon γ from natural killer cells [211]. For example, IL-10, TGF-β, and tryptophan catabolizing enzyme indoleamine 2,3-dioxygenase secreted from them were reported to suppress the proliferation of T cells and change the cytokine secretion profile of T cell subsets [212]. Moreover, the proliferation, differentiation, and chemotactic properties of B cells were impaired by MSCs as well. Except for the immune regulatory effects, MSCs can enhance the restoration of capillary barriers, inhibit bacterial growth, and restore alveolar ATP. All these functions mentioned above might also be effective in COVID-19 infection.

COVID-19 has been the top priority of global healthcare systems since its emergence. There have been more than 160 vaccines in development and more than 60 clinical trials are ongoing, and now, only a few vaccines have been approved [213]. The representative clinical trials of MSC therapy in COVID-19 disease were listed in Table 8. But the rapid mutation of SARS-CoV-2 virus leads to challenges on the effect of the available vaccine. It is an urgent need to develop more universal and stable therapy to reverse or combat. Though no evidence has showed that coronavirus was eliminated completely after stem cell treatments, preliminary results were promising. Diseased patients were more likely to survive the infection after the treatment. The specific primed MSCs were also investigated for COVID-19 treatment [212, 214]. The results will provide a strong foundation for future scientific research and clinical applications for a variety of diseases including pandemic crisis and pulmonary complications. Hopefully, the approaches utilizing MSCs particularly the primed MSCs could be vital for the success of cell therapy in treating COVID-19.

**Table 8:** MSCs therapies for COVID-19 in clinical trials

Study name	NCT number	Starting date	Phase	Key findings/study status
Mesenchymal Stem Cell Therapy for SARS-CoV-2-related Acute Respiratory Distress Syndrome	NCT04366063	April 2020	2–3	Recruiting
UC-MSCs in the treatment of novel coronavirus severe pneumonia	NCT04273646	February 2020	Not applicable	Not yet recruiting
A pilot clinical study on inhalation of MSCs exosomes treating severe novel coronavirus pneumonia	NCT04276987	February 2020	1	Not yet recruiting
UC-MSCs treatment for the 2019-novel coronavirus pneumonia	NCT04269525	February 2020	2	Recruiting
Treatment with MSCs for severe corona virus disease 2019	NCT04288102	February 2020	1–2	Not yet recruiting
MSCs treatment for pneumonia patients infected with 2019 novel coronavirus	NCT04252118	January 2020	1	Recruiting
Nest Cell ®Mesenchymal Stem Cell to Treat Patients with Severe COVID19 Pneumonia	NCT04315987	April 2020	1	Not yet recruiting
Treatment of COVID19 Patients Using Wharton’s Jelly Mesenchymal Stem Cells	NCT04313322	March 2020	1	Recruiting
Novel Coronavirus Induced Severe Pneumonia Treated by Dental Pulp Mesenchymal Stem Cells	NCT04302519	March 2020	Early phase 1	Not yet recruiting
Safety and Efficacy Study of Allogeneic Human Dental Pulp Mesenchymal Stem Cells to Treat Severe COVID19 Patients	NCT04336254	April 2020	1 and 2	Recruiting
Clinical Research of Human Mesenchymal Stem Cells in the Treatment of COVID19 Pneumonia	NCT04339660	February 2020	1 and 2	Recruiting
Bone Marrow-Derived Mesenchymal Stem Cell Treatment for Severe Patients With Coronavirus Disease 2019 (COVID19)	NCT04346368	April 2020	1 and 2	Not yet recruiting
Adipose Mesenchymal Cells for Abatement of SARS CoV-2 Respiratory Compromise in COVID-19 Disease	NCT04352803	April 2020	1	Not yet recruiting
A Clinical Trial to Determine the Safety and Efficacy of Hope Biosciences Autologous Mesenchymal Stem Cell Therapy (HBadMSCs) to Provide Protection Against COVID19	NCT04349631	May 2020	2	Enrolling by invitation
Repair of Acute Respiratory Distress Syndrome by Stromal Cell Administration (REALIST) (COVID19) (REALIST)	NCT03042143	January 2019	1 and 2	Recruiting
Safety and Efficacy of Intravenous Wharton’s Jelly-Derived Mesenchymal Stem Cells in Acute Respiratory Distress Syndrome due to COVID19	NCT04390152	June 2020	1 and 2	Not yet recruiting
Treatment of COVID19 Associated Pneumonia with Allogenic Pooled Olfactory Mucosa-derived Mesenchymal Stem Cells	NCT04382547	May 2020	1 and 2	Not yet recruiting
Clinical Trial to Assess the Safety and Efficacy of Intravenous Administration of Allogeneic Adult Mesenchymal Stem Cells of Expanded Adipose Tissue in Patients with Severe Pneumonia due to COVID19	NCT04366323	April 2020	1 and 2	Not yet recruiting
Study of the Safety of Therapeutic Tx with Immunomodulatory MSC in Adults with COVID19 Infection Requiring Mechanical Ventilation	NCT04397796	June 2020	1	Not yet recruiting
Efficacy and Safety Evaluation of Mesenchymal Stem Cells for the Treatment of Patients with Respiratory Distress to COVID19	NCT04390139	May 2020	1 and 2	Recruiting
Mesenchymal Stem Cells (MSCs) in Inflammation-Resolution Programs of Coronavirus Disease 2019 (COVID19) Induced Acute Respiratory Distress Syndrome	NCT04377334	May 2020	2	Not yet Recruiting
Efficacy and Safety Study of Allogeneic HB-adMSCs for the Treatment of COVID19	NCT04362189	May 2020	3	Not yet Recruiting
Clinical Trial of Allogeneic Mesenchymal Cells from Umbilical Cord Tissue in Patients with COVID19	NCT04366271	May 2020	2	Recruiting
A Randomized, Double-Blind, Placebo-Controlled Clinical Trial to Determine the Safety and Efficacy of Hope Biosciences Allogeneic Mesenchymal Stem Cell Therapy (HBadMSCs) to Provide Protection Against COVID19	NCT04348435	April 2020	2	Enrolling by invitation
Safety and Effectiveness of Mesenchymal Stem Cells in the Treatment of Pneumonia of Coronavirus Disease 2019	NCT04371601	March 2020	2	Active not Recruiting
Use of UC-MSCs for COVID19 Patients	NCT04355728	April 2020	Early Phase 1	Recruiting
Clinical Use of Stem Cells for the Treatment of COVID19	NCT04392778	April 2020	1 and 2	Recruiting
Study of the Safety of Therapeutic Tx with Immunomodulatory MSC in Adults with COVID19 Infection Requiring Mechanical Ventilation	NCT04397796	June 2020	1 and 2	Not yet Recruiting
Efficacy and Safety Evaluation of Mesenchymal Stem Cells for the Treatment of Patients with Respiratory Distress to COVID19	NCT04390139	May 2020	1	Recruiting

## Conclusions

Although MSCs therapies have achieved tremendous advancements over the past decades, substantial challenges remain to be overcome. The main challenges include the immunocompatibility, stability, heterogeneity, differentiation, and migratory capacity. More and more studies are focusing on the attempts to overcome these shortcomings. Although the detailed mechanism of MSCs immunomodulatory effects is still elusive and any attempts to improve MSCs efficacy are still lack of evidence, the preclinical studies are developing rapidly and more standardized clinical trials are wildly carried out. It might be expected that the conversion to canonically registered MSC therapies will flourish with time. The lessons from the current MSCs investigations may provide critical guidance for investigators pursuing further translational processes. With the clarification of MSCs effectors and the emergences of new technologies assisting in-depth studies, MSCs are promising to be proved as effective treatment options for a variety of devastating conditions.

## Data Availability

The material supporting the conclusions of this review is included within the article.

## Abbreviations

AI: Artificial intelligence
ALI: Acute lung injures
ALS: Amyotrophic lateral sclerosis
ARDS: Acute respiratory distress syndrome
BMP: Bone morphogenetic protein
BDNF: Brain-derived neurotrophic factor
CD: Crohn’s disease
CM: Conditioned medium
ECM: Extracellular matrix
EDA: Ectodysplasin
EVs: Extracellular vesicles
FBR: Foreign-body response
FDA: Food and Drug Administration
GDNF: Glial cell line-derived neurotrophic factor
GM-CSF: Granulocyte–macrophage colony-stimulating factor
GMP: Good manufacturing practices
GVHD: Graft-versus-host disease
hBMMSC-EVs: Extracellular vesicles from human bone marrow-derived mesenchymal stromal cells
HGF: Hepatocyte growth factor
HLA: Human leukocyte antigen
hUCMSC-EVs: Extracellular vesicles from human umbilical cord-derived mesenchymal stromal cells
IDO: Indoleamine 2,3-dioxygenase
IGF: Insulin-like growth factor
IL6ST: IL6 signal transducer
MHC: Major histocompatibility complex
MI: Myocardial infarction
MIT: Massachusetts Institute of Technology
MPs: Microparticles
MS: Multiple sclerosis
MSCs: Mesenchymal stromal cells
MVs: Microvesicles
NGF: Nerve growth factor
NTFs: Neurotrophic factors
oGVHD: Ocular graft-versus-host disease
PBMCs: Peripheral blood mononuclear cells
TALEN: Transcription activator like effector nuclease
Trx1: Thioredoxin-1
VCAM1: Vascular cell adhesion molecule-1
VEGF: Vascular endothelial growth factor
ZFNs: Zinc-finger nucleases
